# Case of Congenital Umbilical Hernia Treated by Compression

**Published:** 1884-04

**Authors:** Jordan L. Loyd

**Affiliations:** Surgeon to the Queen’s Hospital, Birmingham


### Selections.

### CASE OF CONGENITAL UMBILICAL HERNIA TREATED BY COMPRESSION.—RECOVERY.

BY JORDAN LLOYD, F.R.C.S. ENG., SURGEON TO THE QUEEN'S HOSPITAL, BIRMINGHAM.

[The Birmingham Medical Review.]

The condition implied by the above title is one occasionally met with in the infant at the time of its birth. It differs materially from the umbilical hernia which develops at a period subsequent to the separation of the cord. In the former the coverings of the rupture consist, to a greater or less extent, of cord tissue proper, and in the latter, of true skin or expanded cicatrix; the former is due to developmental deficiency during intra-uterine life, and the latter to weakness of the abdominal parieties subsequently. The method of treatment in the former is unsettled and usually unsatisfactory, and in the latter, definite and usually successful.

Two cases recently reported, one by Dr. Ronaldson, in the Edinburgh Clinical and Pathological Journal for October 13th, and the other by Mr. Robinson, in the Lancet for September 22d, have led me to place the following on record; because in both of the above, attempts were made to close the abdominal cleft by operative procedure; and in both failure resulted. Dr. Ronaldson's patient recovered from the operation but is reported as still having an umbilical hernia; and Mr. Robinson's died within two hours of operation and fifteen of birth.

A girl three days old was sent to me by my friend, Dr. Leech, on January 20th of last year, when the following notes were taken: "The child is thin and small, and appears to be premature (mother, however, is sure that she carried it to term). She has a swelling covering almost the whole front of the belly, measuring 3½ inches in length, 2½ inches in breadth, and standing out from the parieties as large as an ordinary lemon. The opening into abdomen is two inches in diameter. Three-fourths of an inch of the first part of the tumor is covered by skin, and the rest by expanded cord tissue of a dirty green color and semi-transparent; its outer layer is peeling off. Where this sloughing cord joins the skin a pink line of demarcation has formed, and has gone on to ulceration at two or three small spots. The contents of the tumor cannot be returned into belly; there appears to be insufficient space to contain them. When the child cries the hernia "looks as if it would burst." The child was sent to me for the purpose of having the edges of the abdominal opening refreshed and the parts stitched together. Several of my colleagues saw the case, and agreed that the whole contents of the hernia could not be returned into the already limited abdominal cavity; much less so if this space was still further diminished by the proposed operation. I therefore reduced as much as the belly would contain, and applied a large, thick, somewhat conical pad of dry lint over the remaining swelling, and strapped the infant with emplastrum elemi from its hips to its axillæ. I sent the child away, thinking it must certainly die within a few days. I neither saw nor heard of it until February 22d, nearly five weeks afterwards, when the mother brought it again to hospital. The dressings had not been disturbed since their first application; they were satur ted with a thick, black, viscid material, which smelt disagreeably. On exposing the belly, there was still a protrusion, but only about half an inch in height and barely two inches in diameter; on its summit was a pale circular ulcer exactly the size of a florin. Child throughout had been very well except for frequent vomiting; its bowels had acted regularly and its stools were natural. The sore was dressed with simple ointment, and a pad and strapping applied as before. The case was afterwards dressed once a week, and the sore had healed by March 21st; a small, flat, hernial swelling still remaining. The father was a robust healthy man; the mother delicate. They have a child 3½ years old who "wears irons;" another 2 years unable to walk; and a third died of "consumption of the bowels" at 11 months.

I have read Dr. Ronaldson's case carefully, and think that the protrusion in mine was as large as in his patient. It is somewhat smaller than the measurements given in his text, but about equal to the swelling depicted in his diagram. There seems to be a discrepancy between his description and his figure; for if the former be correct, we must conclude that the child, a few hours old, had an antero posterior abdominal measurement of 10 inches, which, to say the least of it, is unlikely. When referring to the question of treatment, Dr. Ronaldson says—"It was evident that with a hernia of such a size (the sharp-edged, deep, aponeurotic opening in the abdominal wall was from two to two-and-a-half inches in diameter), reducing it and applying a pad and bandage would have been of no service, because, in the first place, the amniotic covering would soon have sloughed, and thereby exposed the child to all the risks of peritonitis; and because, in the second place, it would have been practically hopeless, the amniotic covering being gone, to attempt to prevent wholesale extrusion of the abdominal contents by means of such treatment." He remarks also, that "although cures by pad and bandage, or by ligature, have resulted, they could only have occurred probably when the hernia was of small size, much smaller than that above recorded." My case certainly shows that a well applied compression apparatus is not so useless a treatment as he imagines even in very large hernias. He refers to the operation practiced by himself and Mr. Robinson as simple, easy, effective, and safe. That it is simple and easy I quite agree, but that it is effective and safe is certainly not yet proven. His own case shows it to be ineffective in curing the hernia—the only result which could give it any claim over a simpler and, to my mind, a less dangerous procedure.

The result in the case I have related was a great surprise to myself; I quite expected it would die from peritonitis when the demarking ulceration had extended through to the abdominal cavity. In reference to this apparent liability to peritoneal inflammation from extension of ulceration, Chelius has written that "Experience proves that, if the swelling be properly protected from all external pressure, granulations may be produced after separation of the external covering, and thus the whole part be gradually covered with firm skin and a tendinous expansion." The precise pathological phenomena which occurred beneath the dressing can only be conjectured. I imagine that the ulceration at the edge of skin deepened; that the cord tissue first of all shriveled up under the influence of the overlying dry lint pad; that this necrotic process did not involve its innermost layer; that contraction of the annular area of ulceration followed; that granulations spread inward between the sphacelus and the delicate membrane beneath, thus effectually protecting the abdominal cavity, and that afterwards the sloughy mass softened in the discharges from this ulcerating area and soaked into the superjacent dressings. These changes were favored by the immobility of the abdominal and lower thoracic parieties, secured by the effective strapping. Respiration during this period was necessarily chiefly diaphragmatic.

The success of my case may be attributed to the dry and infrequent dressing and to the immobility of the affected parts. The only objectionable feature throughout the treatment, was the disagreeable odor emanating from the stale dressings. The medical attendant who counseled this first long delay of five weeks, exercised a wise forbearance in so doing, knowing full well the harm that often comes from meddlesome interference in the healing of wounds.

The two usual procedures advocated since long ago in the treatment of congenital umbilical hernia—compression and ligature—will I think give results favorably comparable to what may be obtained by infantile abdominal section. An operation somewhat similar to that practiced by Dr. Ronaldson was successfully performed by Dr. Hamilton nearly 80 years ago. After reducing the hernia, the neck of the sac was ligatured and cut off; the abdominal edges brought together with pins and plaster. Without saying that it is unjustifiable to excise the sac in such cases and to suture the parieties together, I think it will rarely be necessary; and unless it effects a radical cure of the hernia as well as a saving of life, I doubt whether it will succeed in establishing any permanent position in the future over the simpler, safer, and older methods of treatment.

